# Activation of Astrocytes and Microglial Cells and CCL2/CCR2 Upregulation in the Dorsolateral and Ventrolateral Nuclei of Periaqueductal Gray and Rostral Ventromedial Medulla Following Different Types of Sciatic Nerve Injury

**DOI:** 10.3389/fncel.2018.00040

**Published:** 2018-02-19

**Authors:** Petr Dubový, Ilona Klusáková, Ivana Hradilová-Svíženská, Marek Joukal, Pere Boadas-Vaello

**Affiliations:** ^1^Department of Anatomy, Division of Neuroanatomy, Faculty of Medicine, Masaryk University, Brno, Czechia; ^2^Research Group of Clinical Anatomy, Embryology and Neuroscience (NEOMA), Department of Medical Sciences, Universitat de Girona, Girona, Spain

**Keywords:** activated glial cells, CCL2/CCR2, neuroinflammation, periaqueductal gray, rostral ventromedial medulla, nerve injury, neuropathic pain model

## Abstract

Peripheral nerve injuries (PNIs) may result in cellular and molecular changes in supraspinal structures possibly involved in neuropathic pain (NPP) maintenance. Activated glial cells in specific supraspinal subregions may affect the facilitatory role of descending pathways. Sterile chronic compression injury (sCCI) and complete sciatic nerve transection (CSNT) in rats were used as NPP models to study the activation of glial cells in the subregions of periaqueductal gray (PAG) and rostral ventromedial medulla (RVM). Molecular markers for activated astrocytes (glial fibrillary acidic protein, GFAP) and microglial cells (OX42) were assessed by quantitative immunohistochemistry and western blotting. The cellular distribution of CCL2/CCR2 was monitored using immunofluorescence. sCCI induced both mechanical and thermal hypersensitivity from day 1 up to 3 weeks post-injury. Unilateral sCCI or CSNT for 3 weeks induced significant activation of astrocytes bilaterally in both dorsolateral (dlPAG) and ventrolateral PAG (vlPAG) compared to naïve or sham-operated rats. More extensive astrocyte activation by CSNT compared to sCCI was induced bilaterally in dlPAG and ipsilaterally in vlPAG. Significantly more extensive activation of astrocytes was also found in RVM after CSNT than sCCI. The CD11b immunopositive region, indicating activated microglial cells, was remarkably larger in dlPAG and vlPAG of both sides from sCCI- and CSNT-operated rats compared to naïve or sham-operated controls. No significant differences in microglial activation were detected in dlPAG or vlPAG after CSNT compared to sCCI. Both nerve injury models induced no significant differences in microglial activation in the RVM. Neurons and activated GFAP+ astrocytes displayed CCL2-immunoreaction, while activated OX42+ microglial cells were CCR2-immunopositive in both PAG and RVM after sCCI and CSNT. Overall, while CSNT induced robust astrogliosis in both PAG and RVM, microglial cell activation was similar in the supraspinal structures in both injury nerve models. Activated astrocytes in PAG and RVM may sustain facilitation of the descending system maintaining NPP, while microglial activation may be associated with a reaction to long-lasting peripheral injury. Microglial activation via CCR2 may be due to neuronal and astrocytal release of CCL2 in PAG and RVM following injury.

## Introduction

Peripheral nerve injuries (PNIs) due either to trauma, disease or surgical intervention, usually result in neuroplastic changes in the central nervous system (CNS) and pain (Berger et al., 2011). Such changes include the activation of endogenous glial cells of the CNS, like microglia (Streit et al., 1999; Hanisch and Kettenmann, 2007; Graeber and Streit, 2010) and astrocytes (Ridet et al., 1997; Pekny and Nilsson, 2005; Sofroniew and Vinters, 2010; Anderson et al., 2014; Pekny and Pekna, 2014). The activated glial cells facilitate pain neurotransmission and induce the central sensitization of spinal neurons located in the dorsal horn. Briefly, PNI causes the generation of ectopic discharges in the lesioned afferent nerve fibers and their neurons (Omana-Zapata et al., 1997; Woolf and Mannion, 1999; Liu et al., 2000; Schaible, 2007), triggering an increased release of excitatory neurotransmitters and neuromodulators onto spinal cord neurons and glial cells. These changes induce central sensitization, glial cell activation, and hyperexcitability of nociceptive spinal cord neurons that consequently increase their discharges through the ascending pain pathways (Zimmermann, 2001; Schaible, 2007; Latremoliere and Woolf, 2009; Woolf, 2011; Burnstock, 2016).

The cellular and molecular changes described till now have been extensively studied mainly in the dorsal horn of the spinal cord and its circuitry (Vranken, 2009, 2012; Boadas-Vaello et al., 2016), but much less is known about supraspinal changes associated with the induction and maintenance of neuropathic pain (NPP) and the underlying mechanisms. Neuroplastic processes in the spinal cord facilitate the generation and conduction of action potentials by the ascending pathways up to supraspinal structures, contributing to both sensory and pain behavior alterations. The periaqueductal gray (PAG) and rostral ventromedial medulla (RVM) are particularly interesting given their pivotal role in nociceptive modulation (Fields et al., 2006). The PAG has a columnar functional organization with discrete ventrolateral PAG (vlPAG) and dorsolateral PAG (dlPAG) columns that have differences in the antinociceptive effects with respect to their dependence on opiate mechanisms (Lane et al., 2004; Lovick and Bandler, 2005; Eidson and Murphy, 2013; Wilson-Poe et al., 2013) and the spinal projection of sciatic nerve (Keay et al., 1997).

Pre-clinical studies of nerve injury models demonstrated glial activation in PAG (Mor et al., 2010; Ni et al., 2016) and RVM (Wei et al., 2008; Guo et al., 2012) associated with changes in cytokines/chemokines (Wei et al., 2008; Norman et al., 2010; Chu et al., 2012; Guo et al., 2012). Moreover, the chemokine CCL2, also known as monocytes chemoattractant protein 1 (MCP-1), has been demonstrated to play a critical role in NPP facilitation via its preferred receptor, CCR2 (Gao et al., 2009; Jung et al., 2009; Gao and Ji, 2010). It was demonstrated that PNI increases the release of CCL2 in the dorsal horn of the spinal cord (Van Steenwinckel et al., 2011; Clark et al., 2013) triggering the activation of microglial cells (Zhang and De Koninck, 2006; Thacker et al., 2009). The role of CCL2 signaling in supraspinal structures involved in pain modulation has still not been completely elucidated. Reactive microglia cells may synthesize and secrete more inflammatory mediators, which elicit increasing excitability of superficial dorsal horn neurons, and enhance secretion of CCL2 from spinal astrocytes (Clark et al., 2013). Similar processes may occur also in both PAG subregions as well as in the RVM which are involved in the alteration of descending pain modulation.

Overall, despite the available data regarding neuroplastic changes in supraspinal structures associated with nervous system injury-induced pathological pain (Boadas-Vaello et al., 2017), the cellular and molecular processes occurring in these structures as a consequence of PNI are not yet fully understood. Particularly, since distinct subregions of PAG may play different or specific roles in the descending modulation of pain (McMullan and Lumb, 2006; Eidson and Murphy, 2013), it is of interest to investigate whether dlPAG and vlPAG show different pathophysiological responses after PNI, and whether these responses appear in parallel with RVM glial activation, and to do so in distinct PNI models. To this end, the present work was designed to study glial activation in both PAG nuclei and RVM after two models of sciatic nerve injury, namely, sterile chronic constriction injury (sCCI) and complete sciatic nerve transection (CSNT). Moreover, considering the pivotal role of chemokines in CNS sensitization and NPP maintenance, the aim of experiments was also to explore associated chemokine signaling through monitoring the expression of CCL2/CCR2 which could be involved in supraspinal glial cross-talk after sciatic nerve injury.

## Materials and Methods

### Animals and Surgical Procedures

The experiments were performed in 28 adult male rats (Wistar, 200–250 g, Anlab, a.s. Brno, Czech Republic). Animals were kept at 22°C and maintained on a 12 h light/dark cycle under specific pathogen-free conditions in the animal housing facility of the Masaryk University. Sterilized food and water were available *ad libitum*. All surgical treatments were carried out under sterile conditions by the same person in accordance with the European Convention for the Protection of Vertebrate Animals Used for Experimental and Other Scientific Purposes and the protocol was approved by the Animal Investigation Committee of the Faculty of Medicine, Brno, Czech Republic.

Animals were anesthetized by intraperitoneal (i.p.) injection of ketamine (60 mg/kg) and xylazine (7.5 mg/kg) and the right sciatic nerve was exposed at the level of the mid-thigh by blunt dissection and separated from adhering tissue just proximal to its trifurcation. Three ligatures (3-0 Ethicon) were tied around the nerve with 1 mm spacing to reduce nerve diameter by approximately one third of its original diameter in the sCCI group (*n* = 7).

The right sciatic nerve was transected and the proximal stump was tightly ligated and turned back to prevent spontaneous reinnervation in the CSNT group (*n* = 7). The skin of the right paws of CSNT group animals was covered with picric acid solution to prevent autotomy. The right sciatic nerve was only exposed without any lesion in rats (*n* = 7) subjected to a sham operation. Retracted muscles and skin incision were closed with 3-0 silk sutures and the operated rats were left to survive for 3 weeks. Seven intact rats were used as naïve controls.

### Behavioral Tests

Behavioral responses to noxious mechanical (paw withdrawal threshold, PWT) and thermal (paw withdrawal latency, PWL) stimuli were measured in both ipsi- and contralateral hind paws by Dynamic plantar esthesiometer and Plantar test (UGO BASILE), respectively. The location of measurement was in the glabrous skin of the hind paws in the portion of the dermatome innervated by the tibial nerve. Rats were first acclimated in clear Plexiglas boxes for 30 min prior to testing. In the case of thermal hyperalgesia, withdrawal time was measured and the intensity radiance was set to a value of 50. The paws were tested alternately with a 5 min interval between tests. Six PWT and PWL measurements were taken for each paw during each test session 1 day before and 1, 3, 7, 14 and 21 days after operation.

Data for mechanical allodynia and thermal hyperalgesia were expressed as mean ± SD of PWT in grams and PWL in seconds, respectively. All behavioral tests were conducted in a blind manner.

After last behavioral tests at day 21 from operation, animals were sacrificed with a lethal dose of anesthetic and tissue samples were removed for immunohistochemical and western blot analysis.

### Immunohistochemical Staining

Three naïve rats and three rats from the sCCI, CSNT and sham operation groups were deeply anesthetized with a lethal dose of sodium pentobarbital (70 mg/kg body weight, i.p.) and perfused transcardially with 500 ml phosphate-buffered saline (PBS, 10 mM sodium phosphate buffer, pH 7.4, containing 0.15 M NaCl) followed by 500 ml of Zamboni’s fixative (Zamboni and Demartin, 1967).

Brainstem tissue between the superior and inferior colliculi was removed and immersed in Zamboni’s fixative at 4°C overnight. The samples were washed in 20% phosphate-buffered sucrose for 12 h and blocked in Tissue-Tek^®^ OCT compound (Miles, Elkhart, IN, USA). Serial PAG coronal sections (12 μm) from the central part of the superior colliculi to the upper edge of the inferior colliculi corresponding to PAG between 6.72–8.04 mm from the bregma (Paxinos and Watson, 1997) were cut (Leica 1800 cryostat; Leica Microsystems, Wetzlar, Germany). In the same brainstem tissue, coronal sections (12 μm) through the medulla 1 mm from posterior edge of the inferior colliculi corresponding to RVM between −10.8 mm and −11.4 mm from the bregma (Paxinos and Watson, 1997) were also prepared. The sections were collected on gelatin-coated microscopic slides, air-dried and processed for immunohistochemical staining.

Increased expression and extent of GFAP and OX42 immunostaining are widely accepted markers of activated astrocytes (Pekny and Pekna, 2004) and microglial cells (Blackbeard et al., 2007), respectively. Therefore, indirect immunofluorescence staining for GFAP and OX42 was used to detect activation of astrocytes and microglial cells in PAG and RVM after sciatic nerve injury. Briefly, the sections were washed with PBS containing 0.05% Tween 20 (PBS-TW20) and 1% bovine serum albumin for 10 min, and then treated with 3% normal donkey serum in PBS-TW20 for 30 min. The sections were incubated with 50 μl of mouse monoclonal anti-CD11b/c antibody (OX42), rabbit polyclonal anti-glial fibrillary acidic protein (GFAP) or rabbit polyclonal anti-CCL2 antibody in a humid chamber at room temperature (21–23°C) for 12 h to identify activated microglial cells, astrocytes or CCL2 expression, respectively. The immunoreaction was visualized by treatment with FITC- or TRITC-conjugated, affinity purified, donkey anti-mouse or anti-rabbit secondary antibodies for 90 min at room temperature. Cell nuclei were stained using Hoechst 33342 (Sigma, St. Louis, MO, USA) and the sections were mounted in Vectashield aqueous mounting medium (Vector Laboratories, Burlingame, CA, USA).

A set of PAG and RVM sections was double immunostained to detect cell types producing CCL2 or expressing CCR2. The sections were incubated with rabbit or chicken anti-GFAP antibody to detect activated astrocytes, with mouse monoclonal OX42 for co-localization in activated microglial cells and polyclonal or monoclonal NeuN antibody to identify neurons. The first incubations were combined with immunostaining with rabbit polyclonal anti-CCL2 antibody or goat polyclonal anti-CCR2 antibody. The binding of primary antibodies were visualized by appropriate FITC-, FITC- or AlexaFluor 647-conjugated secondary antibodies (Table 1).

**Table 1:** List of primary and secondary antibodies used for immunofluorescence detection.

	Antibody	Source	Product	Dilution
GFAP	pAb	Rabbit	Dako	1:250
GFAP	pAb	Chicken	Abcam	1:500
OX42	mAb	Mouse	Serotec	1:50
NeuN	pAb	Rabbit	Millipore	1:500
NeuN	mAb	Mouse	Chemicon	1:500
CCL2	pAb	Rabbit	Serotec	1:250
CCR2	pAb	Goat	ThermoFisher	1:100
Anti-chicken	pAb	Goat	Abcam	1:200
Anti-rabbit	pAb	Donkey	Millipore	1:400
Anti-mouse	pAb	Donkey	Millipore	1:400
Anti-mouse	pAb	Goat	Millipore	1:400

*pAb, polyclonal antibody; mAb, monoclonal antibody*.

Control sections were incubated by omitting the primary antibodies (data not shown). Cell nuclei were stained using Hoechst 33342 and sections were analyzed using a Nikon Eclipse NI-E epifluorescence microscope equipped with a Nikon DS-Ri1 camera driven by NIS-Elements software (Nikon, Prague, Czech Republic).

### Image Analysis

At least 12 sections (separated from one another by an interval of about 80 μm) of PAG or RVM for each animal were selected for image analysis. Immunopositive area for OX42 or GFAP was measured using the NIS-elements image analysis system (Laboratory Imaging Ltd., Prague, Czech Republic). Briefly, the area of interest (40,000 μm^2^ for PAG; 70,000 μm^2^ for RVM) was placed over the dlPAG and vlPAG) or over RVM and the GFAP- or OX-42-immunostained structures were detected by a thresholding technique after subtraction of background. The boundaries of dlPAG and vlPAG columns were defined according to the rat PAG map (Paxinos and Watson, 1997) and other anatomical criteria (Keay and Bandler, 2001). The area of immunostaining for OX42 or GFAP was related to the area of interest and expressed as the mean of relative area (%) ± SD.

### Western Blot Analysis

Naïve, sham-, sCCI- and CSNT-operated rats were used for western blot analysis (four rats for each group). Rats were killed with CO_2_, decapitated and the brainstem was removed. A 2 mm thick coronal slice of the mesencephalon was rapidly removed at the position from the central part of the superior colliculi to the upper edge of the inferior colliculi corresponding to PAG between 6.72 mm and 8.04 mm from the Bregma (Paxinos and Watson, 1997). Radial segments corresponding approximately with ipsilateral and contralateral dlPAG and vlPAG were dissected under a stereomicroscope (see Figure 2E) according to the boundaries defined using anatomical criteria (Keay and Bandler, 2001).

**Figure 1:**
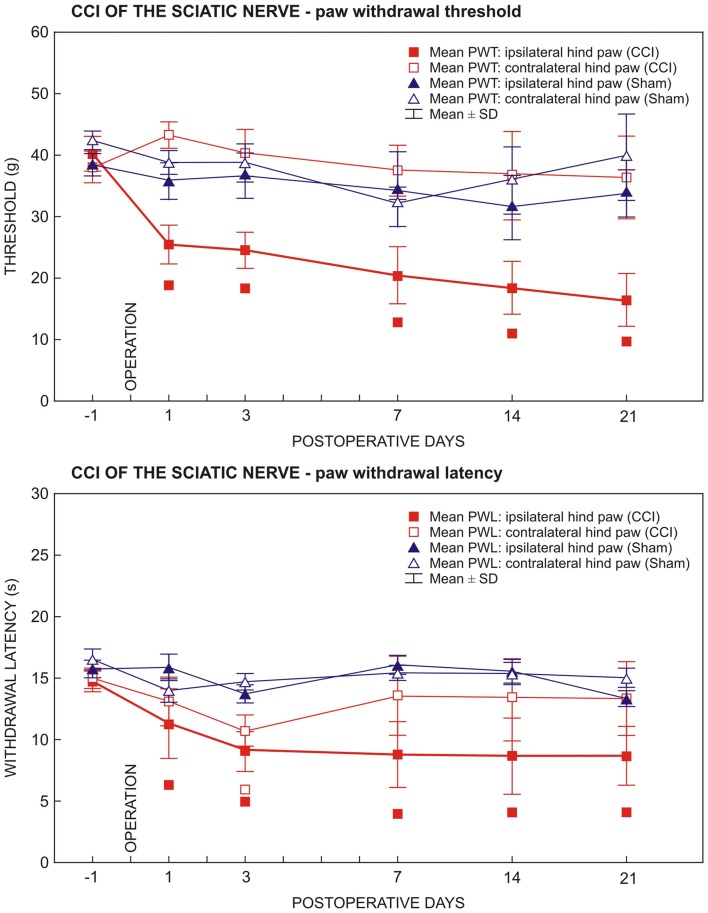
Results of behavioral tests in rats operated upon to create unilateral sterile chronic compression injury (sCCI) of the sciatic nerve and sham-operated rats. Development and maintenance of paw withdrawal threshold (PWT) and paw withdrawal latency (PWL) was measured in the ipsilateral hind paws of sCCI-operated rats indicating mechanical allodynia and thermal hyperalgesia, respectively. Transient thermal hyperalgesia was measured bilaterally 3 days following sCCI. Data are expressed as mean ± SD of PWT in grams and PWL in seconds for mechanical allodynia and thermal hyperalgesia, respectively. Symbols below the curves indicate a statistically significant difference (*p* < 0.05) compared to sham-operated rats.

**Figure 2:**
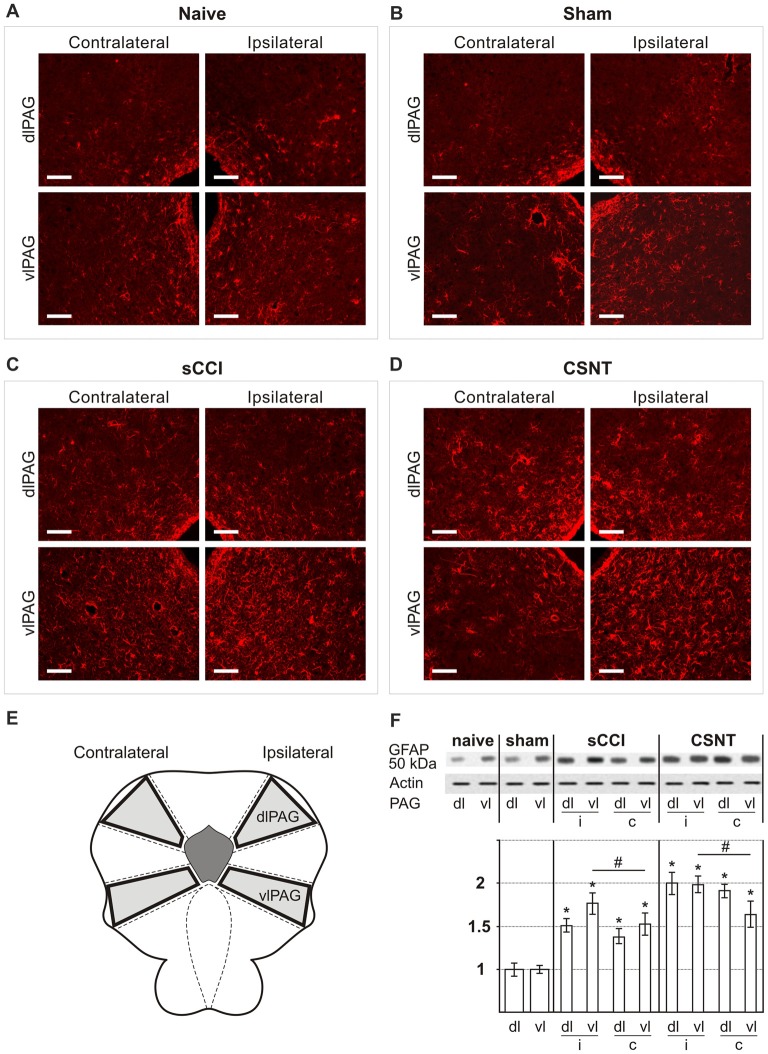
Representative sections through dorsolateral periaqueductal gray (dlPAG) and ventrolateral PAG (vlPAG) of naïve **(A)**, sham- **(B)**, sCCI- **(C)** and complete sciatic nerve transection (CSNT)-operated **(D)** rats immunostained for glial fibrillary acidic protein (GFAP). The figures show a bilateral increase in the area containing activated GFAP+ astrocytes in both dlPAG and vlPAG after sCCI or CSNT for 3 weeks. Scale bars = 80 μm. **(E)** Schematic representation of the location of dlPAG and vlPAG and their sampling for western blot analysis. **(F)** The top panel shows representative western blot of GFAP protein in PAG of naïve and sham-operated rats and 3 weeks after sCCI or CSNT. (i) indicates ipsilateral and (c) contralateral segments of dorsolateral (dl) and ventrolateral (vl) PAG. Graph below the blot illustrates density data obtained from three blots after normalization to actin, expressed as fold-change relative to those of sham-operated rats (set as 1). *Indicates a statistically significant difference (*p* < 0.05) compared to the value from sham-operated rats; # indicates a statistically significant difference (*p* < 0.05) between the values from ipsilateral and contralateral vlPAG.

**Figure 3:**
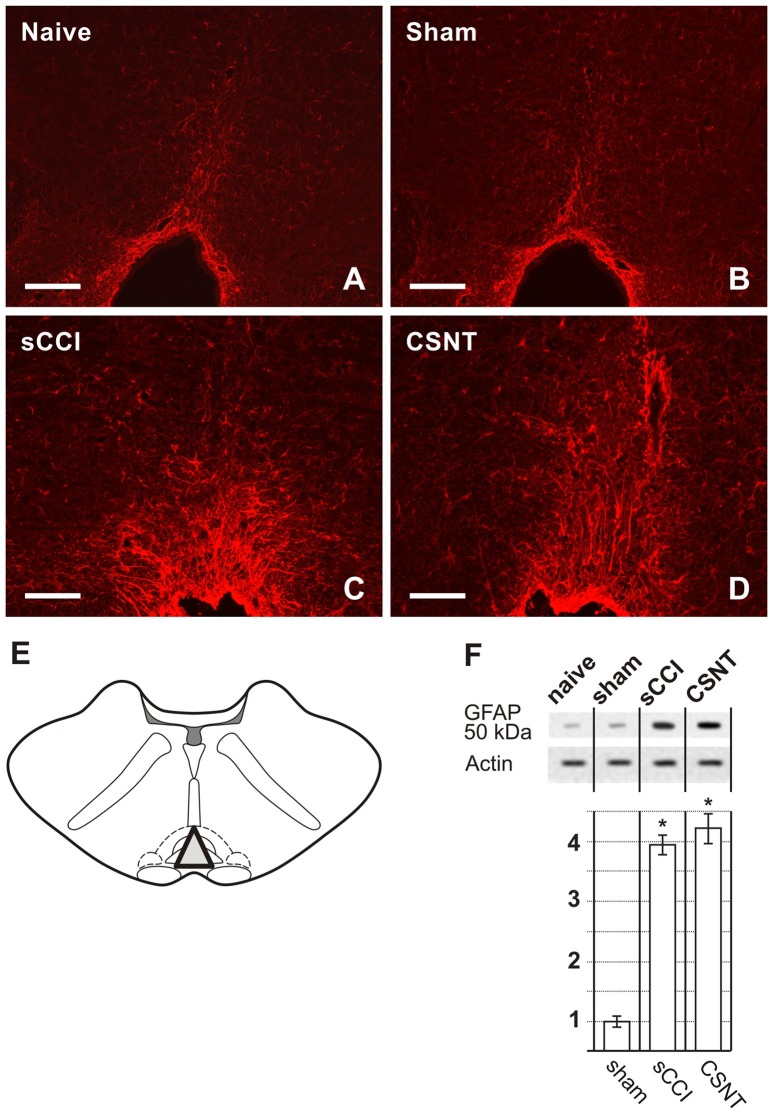
Representative medullary sections showing rostral ventromedial medulla (RVM) from naïve and sham-operated controls **(A,B)** and 3 weeks after sCCI or CSNT **(C,D)**. The pictures illustrate increased intensity and expansion of GFAP immunostaining in RVM after sCCI and CSNT in comparison to RVM from naïve or sham-operated rat. Scale bars = 90 μm. **(E)** The drawing illustrates dissection of RVM samples for western blots including the nucleus raphe magnus and gigantocellularis pars alpha (triangle). **(F)** The top panel illustrates representative western blot of GFAP protein in RVM of naïve and sham-operated rats and 3 weeks after sCCI or CSNT. *Indicates a statistically significant difference (*p* < 0.05) compared to the value from sham-operated rats.

A 2 mm coronal slice was cut though the medulla, 1 mm from posterior edge of the inferior colliculi corresponding approximately to 1 mm from the interaural line. This section corresponds to the RVM between −10.8 mm and −11.4 mm from the bregma (Paxinos and Watson, 1997). A tissue triangle was then dissected under a stereomicroscope to isolate the RVM area, including the nucleus raphe magnus, gigantocellularis, and gigantocelularis pars alpha (see Figure 3E). The RVM samples were not divided into ipsi- and contra-lateral sides because no differences were seen during image analysis of immunostained sections. Tissue samples were collected, frozen in liquid nitrogen, and stored at −80°C until further processing.

The PAG and RVM tissue samples of individual animals were homogenized in PBS containing 0.1% Triton X-100 and protease inhibitors (LaRoche, Switzerland), and centrifuged at 15,000 *g* for 20 min at 4°C. Protein concentration was measured in the tissue supernatant by Nanodrop ND-1000 (Thermo Scientific) and normalized to the same levels. Each sample, containing 50 μg of protein, was separated by SDS-polyacrylamide gel electrophoresis (Wei et al., 2008) and transferred to nitrocellulose membranes by electroblotting (BioRad).

The membranes were blocked with 1% BSA in PBST (3.2 mM Na_2_HPO_4_, 0.5 mM KH_2_PO_4_, 1.3 mM KCl, 135 mM NaCl, 0.05% Tween 20, pH 7.4) for 1 h and incubated with mouse monoclonal anti-CD11b/c antibody (OX42; 1:50, AbD Serotec, Kidlington, UK) or rabbit polyclonal anti- GFAP (1:250, Dako, Glostrup, Denmark) overnight. Blots was washed in PBST and incubated with peroxidase-conjugated anti-mouse or anti-rabbit secondary antibodies (Sigma, 1:1000) at room temperature for 1 h. Equal loading of proteins was confirmed by b-actin staining. Protein bands were visualized using the ECL detection kit (Amersham) on an LAS-3000 chemiluminometer reader (Bouchet Biotech) and analyzed using densitometry image software. No differences were measured between samples of naïve and sham-operated rats, therefore, OX42 and GFAP protein data after normalization to actin were expressed as fold change relative to sham PAG or RVM, which were set to 1.

### Statistical Analyses

Behavioral data among groups were evaluated using two-way analysis of variance (ANOVA) with repeated measurements. One-way ANOVA with Student–Newman–Keuls *post hoc* test was used for comparison at each time point and *p* values less than 0.05 were considered to be significant. Statistical differences between data for staining area and western blot analysis were tested using a Mann-Whitney U-test (*p* < 0.05). All statistical analyses were performed using STATISTICA 9.0 software (StatSoft, Inc., Tulsa, OK, USA).

## Results

### Behavioral Tests

All rats operated on to create sCCI of the sciatic nerve displayed as signs of NPP, decreased thresholds of mechanical allodynia (PWT) and withdrawal latencies of thermal hyperalgesia (PWL) restricted to the hind paws ipsilateral to the nerve ligatures. Decreased PWT and PWL was induced at day 1 and maintained throughout the period of survival up to 3 weeks when compared with sham- operated rats. Hind paws contralateral to sCCI did not exhibit statistically significant changes of PWT and PWL when compared with 1 day before operation or with hind paws of sham-operated rats (Figure 1). In hind paws of sham-operated animals, no significant changes in PWT and PWL data were measured compared to 1 day before operation. No autotomy was found in animals of the CSNT group.

### Activation of Astrocytes in PAG and RVM after Chronic Compression Injury Compared to Complete Transection

Increased immunostaining for GFAP is frequently used to detect activation of astrocytes following various types of nervous system injury (Pekny and Pekna, 2004). We found a significantly larger GFAP+ area corresponding to astrocytes in vlPAG than dlPAG in both naïve and operated rats. Sciatic nerve injury by sCCI or CSNT for 3 weeks induced increased GFAP intensity and a significant enlargement of GFAP+ area bilaterally in both dlPAG and vlPAG when compared to naïve or sham-operated controls. In addition, CSNT gives rise to a larger GFAP+ area in bilateral dlPAG and only ipsilateral vlPAG when compared with sCCI. However, a significantly larger GFAP+ area was seen in vlPAG of the ipsilateral than the contralateral side (Figures 2A–D, Table 2).

**Table 2:** Percentage of glial fibrillary acidic protein (GFAP+) area ±SD in rostral ventromedial medulla (RVM) and dorsolateral (dl) and ventrolateral (vl) periaqueductal gray (PAG) of naïve rats as well as in dlPAG and vlPAG of ipsilateral (ipsi) and contralateral (contra) sides from sham-operated rats and rats with sterile chronic compression injury (sCCI) and complete sciatic nerve transection (CSNT) for 3 weeks (*n* = 6 for each group).

	Naive	Sham		sCCI		CSNT	
		ipsi	contra	ipsi	contra	ipsi	contra
dlPAG	4.3 ± 1.8	5.1 ± 2.6	5.3 ± 2.8	8.6 ± 2.5*	7.9 ± 2.3*	12.5 ± 1.8*^‡^	11.3 ± 2.4*^‡^
vlPAG	10.3 ± 2.2^+^	10.8 ± 2.9^+^	10.9 ± 2.4^+^	17.1 ± 2.2^+^*^†^	13.8 ± 2.8^+^*	23.6 ± 3.5^+^*^†‡^	15.2 ± 2.8^+^*
RVM	3.8 ± 1.0	4.1 ± 1.9		17.5 ± 1.3*		20.8 ± 2.1*^‡^	

^+^Significant difference (*p* < 0.05) compared to dlPAG. *Significant difference (*p* < 0.05) compared to Naïve or Sham. ^†^Significant difference (*p* < 0.05) compared to contralateral side. ^‡^Significant difference (*p* < 0.05) compared to sCCI.

Sections through RVM prepared from the same rats surviving for 3 weeks with sCCI or CSNT also displayed an increase in GFAP intensity and robust enlargement of the GFAP immunopositive area in comparison to RVM sections of naïve or sham-operated rats (Figures 3A–D). Similarly to PAG, CSNT gives rise to a larger GFAP+ area than in the same structures of animals subjected to sCCI (Table 2).

The increased activation of astrocytes in PAG and RVM after sCCI and CSNT detected by image analysis of GFAP immunofluorescence areas was confirmed by western blot analysis of the GFAP protein (Figures 2F, 3F).

### Microglia Activation in PAG and RVM after Chronic Compression in Comparison to Transection

**Figure 4:**
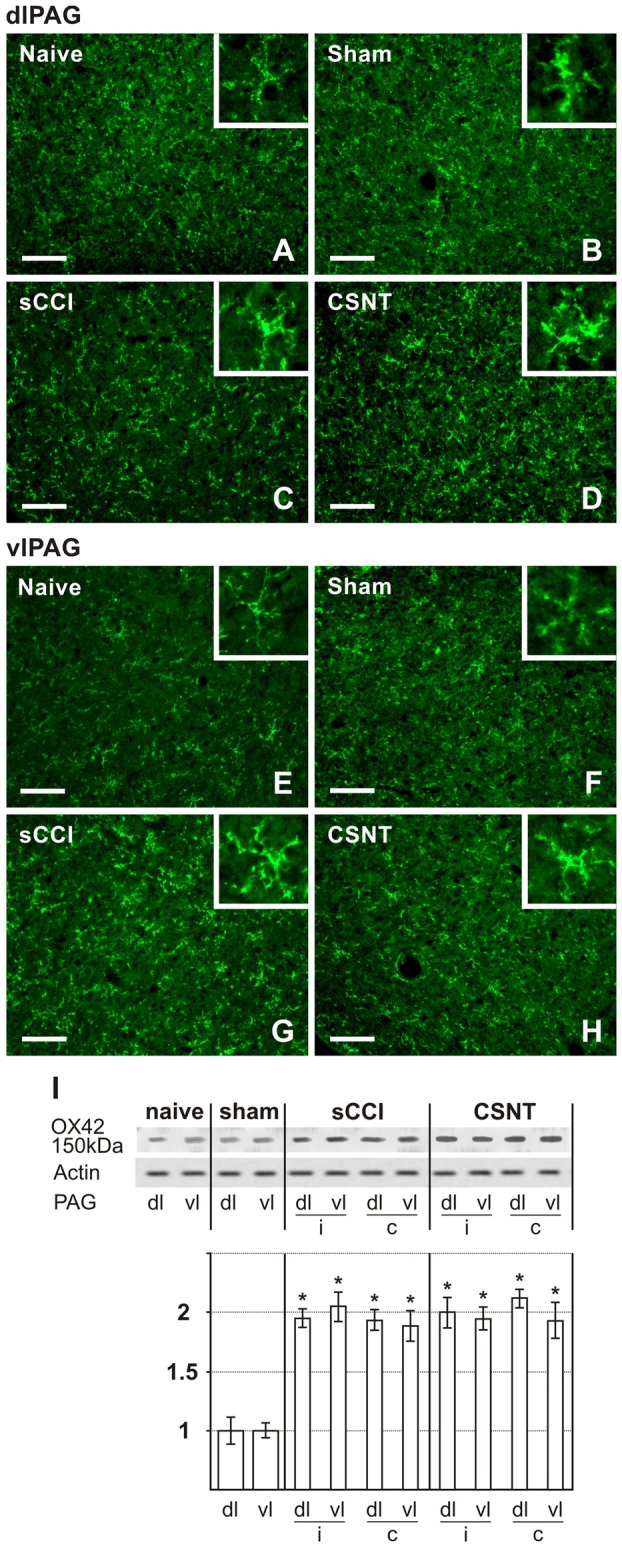
Representative sections illustrating OX42 immunofluorescence staining in dlPAG **(A–D)** and vlPAG **(E–H)** of naïve, sham-, sCCI- and CSNT-operated rats. Insets show the typical morphological types of OX42+ microglial cells from the respective animal groups. Scale bars = 90 μm. **(I)** Western blot analysis of OX42 (CD11b/c) protein. The top panel illustrates representative western blot in PAG of naïve and sham-operated rats and rats 3 weeks after sCCI or CSNT. (i) indicates ipsilateral and (c) contralateral segments of dorsolateral (dl) and ventrolateral (vl) PAG. Graph below the blot illustrates density data obtained from three blots after normalization to actin, expressed as fold-change relative to those of sham-operated rats (set as 1). *Indicates a statistically significant difference (*p* < 0.05) compared to the value from sham-operated rats.

Activated microglial cells were identified by increased immunofluorescence staining for CD11b, detected using the antibody OX42 and by changes in their shape. Weak OX42 immunofluorescence and microglial cells with limited ramification occupied a similar proportion of immunostained areas in both vlPAG and dlPAG of naïve and sham-operated animals. The OX42 immunoreactive area indicating activated microglial cells was remarkably larger in vlPAG and dlPAG of both sides in PAG sections from sCCI- and CSNT-operated rats when compared to naïve or sham-operated ones. In contrast to GFAP+ astrocytes, no significant expansion of the OX42 immunoreactive area was detected in either dlPAG or vlPAG after CSNT when compared to sCCI of the sciatic nerve (Figures 4A–H, Table 3). Besides increased immunostaining intensity and enlargement of the OX42+ area, microglial activation was recognized by their changed morphology. In contrast to only a few processes in sections from naïve and sham-operated rats, activated microglial cells in PAG displayed more vigorous ramification along with upregulation of the constitutively expressed marker OX42 (Figures 4A–H, insets).

**Table 3:** Percentage of OX-42+ area ±SD in RVM and dorsolateral (dl) and ventrolateral (vl) PAG of naïve rats as well as in dlPAG and vlPAG of ipsilateral (ipsi) and contralateral (contra) sides from sham-operated rats and rats with sCCI and CSNT for 3 weeks.

	Naive	Sham		sCCI		CSNT	
		ipsi	contra	ipsi	contra	ipsi	contra
dlPAG	6.9 ± 0.9	7.2 ± 1.5	7.0 ± 1.2	13.1 ± 1.8*	12.9 ± 2.6*	13.9 ± 2.5*	13.2 ± 3.7*
vlPAG	7.9 ± 2.7	8.3 ± 2.6	8.1 ± 2.9	13.4 ± 2.4*	12.3 ± 3.7*	13.2 ± 2.4*	12.6 ± 2.2*
RVM	0.7 ± 0.2	0.8 ± 0.2		3.6 ± 1.2*		3.4 ± 0.7*	

*Significant difference (p < 0.05) compared to Naïve or Sham (n = 6 for each group).

**Figure 5:**
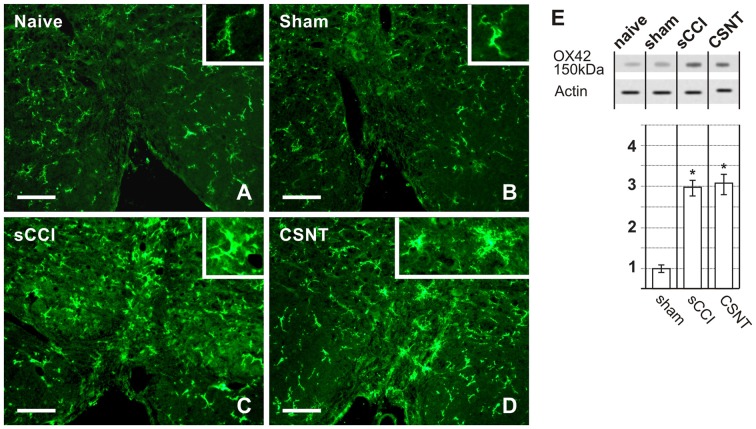
Representative medullary sections showing RVM from naïve and sham-operated controls **(A,B)** and rats with sCCI or CSNT **(C,D)**. The pictures illustrate increased intensity and enlargement of OX42 immunostaining after sCCI and CSNT when compared to RVM from naïve or sham-operated rats. Insets show the typical morphological types of OX42+ microglial cells from the respective animal groups. Scale bars = 40 μm. **(E)** Western blot analysis of OX42 (CD11b/c) protein. The top panel illustrates representative western blot in RVM of naïve and sham-operated rats and rats 3 weeks after sCCI or CSNT. Graph below the blot illustrates density data obtained from three blots after normalization to actin, expressed as fold-change relative to those of sham-operated rats (set as 1). *Indicates a statistically significant difference (*p* < 0.05) compared to the value from sham-operated rats.

Increased OX42 immunostaining intensity and enlargement of the immunopositive area compared to RVM sections of naïve or sham-operated rats was also observed in sections through RVM prepared from the same brainstems as for OX42 analysis in PAG. No significant increase in OX42+ areas was found in RVM after CSNT than sCCI of the sciatic nerve (Figures 5A–D, Table 3). Activated microglial cells in RVM after both types of sciatic nerve injury were not only highly ramified but some were also transformed morphologically to “bushy” type microglia (Figures 5A–D, insets).

Increased activation of microglial cells in PAG and RVM after sCCI and CSNT detected by image analysis of OX42 immunofluorescence areas was confirmed by western blot analysis of the OX42 protein (Figures 4I, 5E).

### Neurons and Activated GFAP+ Astrocytes Display Immunostaining for CCL2 While Activated OX42+ Microglial Cells Are Immunopositive for CCR2 in Both PAG and RVM after Sciatic Nerve Injury

**Figure 6:**
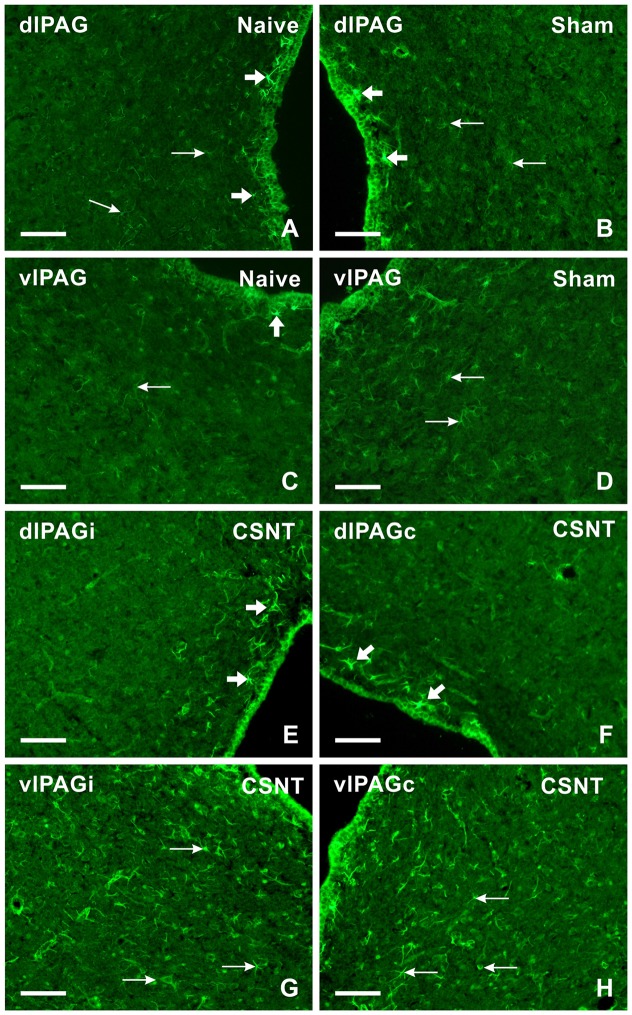
Representative sections showing immunofluorescence staining for CCL2 in dlPAG and vlPAG of naïve **(A,C)**, sham- **(B,D)** and CSNT-operated **(E–H)** rats. Weak CCL2 immunofluorescence was observed in the cells of both dlPAG and vlPAG from naïve and sham-operated controls (arrows in **A–D**). Only some cells located close to the ependymal layer displayed intense CCL2 immunostaining (broad arrows in **A–D**). Sciatic nerve injury for 3 weeks induced increased CCL2 immunofluorescence intensity in the cells (arrows) mainly in vlPAG of both ipsilateral (vlPAGi) and contralateral (vlPAGc) sides **(G,H)**. Sections through dlPAG of the same animal **(E,F)** displayed distinct CCL2 immunofluorescence in a larger number of cells close to the ependymal layer (broad arrows) compared to naïve and sham-operated animals. Scale bars = 80 μm.

**Figure 7:**
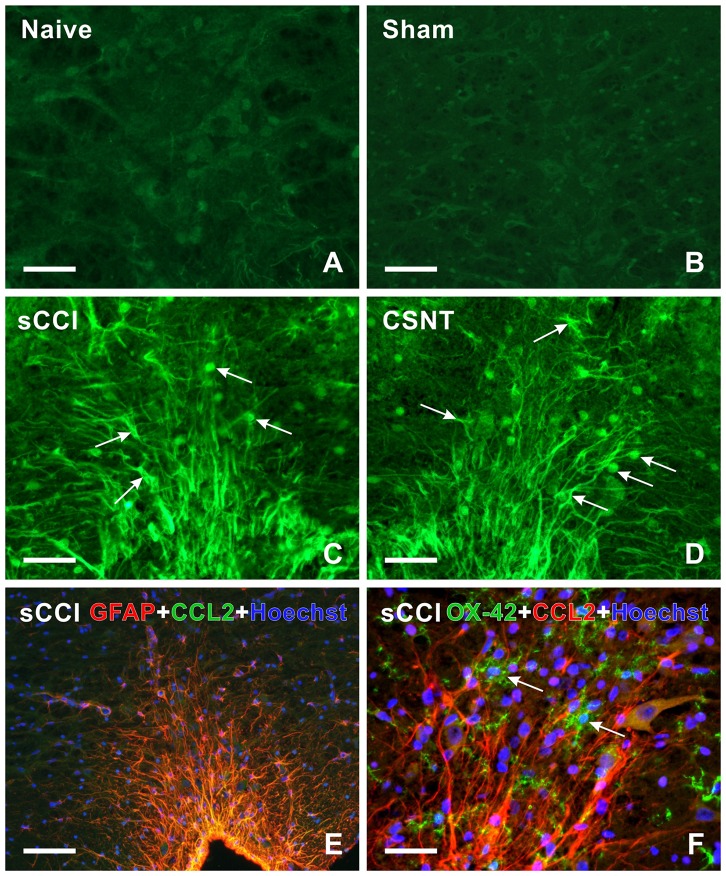
**(A–D)** Representative medullary sections showing RVM from naïve **(A)** and sham-operated **(B)** controls as well as rats 3 weeks from sCCI **(C)** or CSNT **(D)** after immunostaining for CCL2. Compared to RVM of naïve and sham-operated rats **(A,B)**, CCL2 immunofluorescence intensity was significantly increased in cells (arrows) of RVM from rats with injured sciatic nerve **(C,D)**. **(E,F)** Representative pictures of double immunofluorescence for GFAP or OX42 and CCL2 in RVM after sCCI of the sciatic nerve for 3 weeks. **(E)** The section was double immunostained for GFAP (red) and CCL2 (green). The merged picture shows expression of CCL2 protein in all GFAP+ astrocytes of RVM; nuclei (blue) were stained with Hoechst. **(F)** The section was double immunostained for OX42 (green) and CCL2 (red). The merged picture shows the absence of CCL2 protein in OX42+ microglial cells (arrows); nuclei (blue) were stained with Hoechst. Scale bars = 90 μm in **(B,E)**; 40 μm in **(A,C,D,F)**.

Sections through PAG from naïve and sham-operated rats show only weak CCL2 immunofluorescence in the cells present in the dlPAG and vlPAG columns. However, some cells in the narrow region at the Sylvian canal (aqueduct) displayed a higher intensity of CCL2 immunofluorescence than in the dlPAG and vlPAG area (Figures 6A–D). Sciatic nerve injury by sCCI and CSNT for 3 weeks induced a bilateral increase of CCL2 immunofluorescence intensity in cells of vlPAG and ipsilateral dlPAG, while the cells of contralateral dlPAG showed an intensity like that of naïve or sham-operated rats. Intense immunofluorescence intensity of CCL2 was found in the cells of vlPAG than dlPAG 3 weeks after sciatic nerve injury (Figures 6E–H). RVM sections of naïve and sham-operated rats displayed very faint CCL2 immunoreaction, but the intensity increased in the cells 3 weeks after sCCI or CSNT (Figures 7A–D).

**Figure 8:**
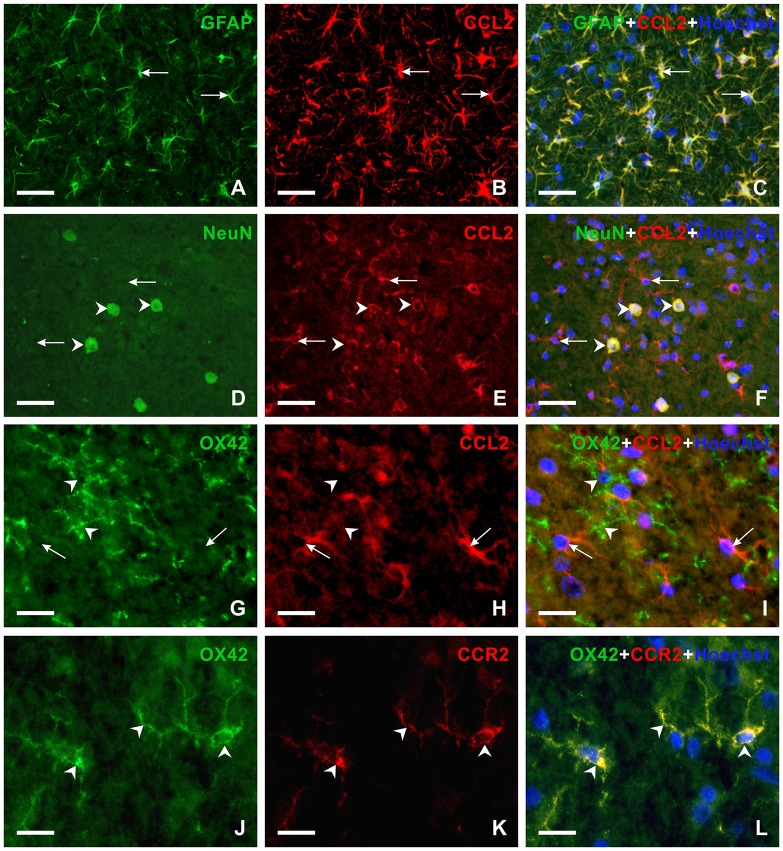
Double immunofluorescence staining in vlPAG of rats 3 weeks from sCCI. **(A–F)** Sections illustrating double immunofluorescence staining for CCL2 and GFAP or NeuN as markers of activated astrocytes or neurons, respectively. The merged pictures **(C,F)** show CCL2 protein in activated astrocytes (arrows) and neurons (arrowheads). **(G–L)** Double immunofluorescence staining for OX42 as a marker for microglial cells and CCL2 **(G–I)** or CCR2 **(J–L)**. The merged picture **(I)** shows the absence of CCL2 protein in microglial cells. Compare CCL2 positive astrocyte-like cells (arrows) with the absence of CCL2 immunoreaction in microglial cells (arrowheads). However, OX42+ microglial cells displayed CCR2 immunostaining (arrowheads in **L**). Scale bars = 40 μm for **(A–F)**; 75 μm for **(G–I)**; 15 μm for **(J–L)**.

To reveal the cellular origin of CCL2 protein, one set of sections were double immunostained for CCL2 and GFAP as marker of activated astrocytes or NeuN, a molecular marker of neurons. Double immunostaining showed the location of CCL2 protein in all GFAP+ astrocytes as well as NeuN+ neurons of PAG and RVM (Figures 7E, 8A–F), while OX42+ microglial cells were free of any CCL2 immunofluorescence (Figures 7F, 8G–I). The same cellular distribution of increased immunostaining for CCL2 was observed in PAG and RVM from animals subjected to both sCCI and CSNT for 3 weeks. Immunostaining for CCR2, a receptor of CCL2, was observed only in OX42+ microglial cells of both PAG and RVM (Figures 8J–L), but no CCR2 immunoreaction was found in GFAP+ astrocytes.

## Discussion

The supraspinal modulatory system is differentially involved in descending facilitatory and inhibitory pathways to influence transmission of nociceptive inputs from the dorsal horn of the spinal cord to the upper structures of the CNS (Heinricher et al., 1989; Porreca et al., 2002). A fine equilibrium between the opposing descending controls exists under normal conditions. Sensitization may durably alter this balance in favor of facilitations that consequently give rise to a NPP state. The RVM and PAG of the brainstem are the principal (and the best studied) structures of the endogenous modulatory system that alter spinal dorsal horn processing of sensory input (Heinricher et al., 2009). Despite a growing body of evidence indicating a role for PAG and RVM in descending pain modulation, the precise underlying cellular and molecular mechanisms involved in descending facilitation and inhibition of the dorsal horn remain elusive.

Several animal models based on sciatic nerve injury are used to investigate NPP mechanisms and test novel analgesics. The chronic constriction injury of the sciatic nerve is most frequent model e included transection. The original CCI model created by four ligatures of chromic gut (4–0) loosely tied around the sciatic nerve results in inflammatory swelling with subsequent compression of the nerve (Bennett and Xie, 1988). However, Wallerian degeneration distal to traumatic nerve injury is considered to be neuroinflammation, and it is impossible to distinguish between nerve inflammation induced by chromic gut (Maves et al., 1993; Clatworthy et al., 1995) and proper inflammatory reactions as a consequence of Wallerian degeneration (Klusáková and Dubový, 2009). Therefore, we used sCCI of the sciatic nerve to study NPP and glial activation induced by traumatic nerve injury when damaged axons are mixed with spared ones influenced with inflammatory mediators produced only by Wallerian degeneration (Dubový, 2011). The CCI model is associated with symptoms of chronic nerve compression (Bennett and Xie, 1988), while the CSNT model is rather an experimental model to study neuropathic symptoms, such as “anesthesia dolorosa”, pain with reference to the area in the absence of any sensory signals (Devor, 1994). In the present study, we used the CSNT model primarily to compare glial reactions after total and partial (sCCI) nerve disconnection.

It is well-known that sensitization of the CNS at different levels by activation of glial cells can contribute to the development and/or maintenance of chronic pain states after a PNI (Suzuki et al., 2004). Most experimental results relating to the role of glial activation in induction and maintenance of NPP were observations made in the dorsal horn of the spinal cord (Blackbeard et al., 2007; Milligan and Watkins, 2009). Less attention has been paid to the activation of glial cells in supraspinal structures. For example, activated astrocytes were found in RVM after unilateral CCI of the rat infraorbital nerve (Wei et al., 2008) and in PAG following CCI of the sciatic nerve (Mor et al., 2010, 2011). In contrast to these separate studies in PAG and RVM of different experimental models with varying times of survival, we used the sCCI and CSNT models for 3 weeks to compare long-lasting activation of astrocytes and microglial cells in both RVM and PAG depending on the extent of nerve injury.

### Activation of Glial Cells in Ventrolateral and Dorsolateral Subregions of PAG

The vlPAG with the spinal projection of sciatic nerve (Keay et al., 1997) has a significant role in descending control of noxious afferentation via connections with RVM (Eidson and Murphy, 2013). In addition to descending control, vlPAG columns have projections to many CNS structures critical for the normal expression of sleep-wake cycles and social behaviors (Monassi et al., 2003; Mor et al., 2011). An increased number of GFAP+ astrocytes in vlPAG was mainly detected in a subset of rats which displayed both NPP and disability following CCI of the sciatic nerve (Mor et al., 2011). In contrast, dlPAG column has projections primarily to the dorsolateral pons and the ventrolateral medulla that are implicated in autonomic control (Cameron et al., 1995). Moreover, descending control from dlPAG has different effects on nociceptive reflexes evoked by activation of C- and A-delta fibers (McMullan and Lumb, 2006).

Sciatic nerve injury by sCCI or CSNT for 3 weeks induced activation of astrocytes bilaterally in both dlPAG and vlPAG when compared to naïve or sham-operated control rats. Significantly more extensive activation of astrocytes was observed in vlPAG on the ipsilateral than the contralateral side in both models of unilateral sciatic nerve injury that can be related with the spinal projection of sciatic nerve (Keay et al., 1997). In contrast, ipsilateral dlPAG displayed a more extensive activation of astrocytes when compared to the contralateral column but this change was not significant. Our results also revealed a higher induction of astroglial activation in PAG columns by CSNT than sCCI indicating a dependence on the extent of the sciatic nerve injury.

In contrast to astrocytes, activation of microglial cells was similar in dlPAG and vlPAG of both sides in the sCCI and CSNT models of the sciatic nerve injury. This suggests that signaling leading to microglial activation is maintained bilaterally in both PAG columns 3 weeks after different types of sciatic nerve injury (sCCI and CSNT).

Taken together, these findings of activated astrocytes and microglial cells in vlPAG after sciatic nerve injury suggest a role for activated glial cells in the modulation of activity of this PAG column with respect to descending inhibition or facilitation of nociceptive input following nerve injury (Ni et al., 2016). It is clinically important that vlPAG and its descending projections to RVM comprise a neural circuit for opioid-mediated analgesia (Lane et al., 2004; Eidson and Murphy, 2013; Wilson-Poe et al., 2013). Increased activation of microglial cells and astrocytes in vlPAG was observed only in those animals made tolerant to morphine (Eidson and Murphy, 2013).

Unilateral sciatic nerve injury (sCCI and CSNT) induced bilateral activation of astrocytes and microglial cells in both dlPAG and vlPAG. These results are probably related with the bilateral projections from the spinal cord to PAG responses of which appear to be predominantly bilateral and non-somatotopic (Yezierski and Mendez, 1991; Jones et al., 2003).

### Activation of Glial Cells in RVM

The RVM provides the major common output of descending modulatory system that is critical in the maintenance of chronic pain states (Pertovaara et al., 1996; Porreca et al., 2002; Dubner and Ren, 2004; Gebhart, 2004; Vanegas and Schaible, 2004; Vera-Portocarrero et al., 2006; Bee and Dickenson, 2007). The RVM is a relay in the pathways from both vlPAG and dlPAG columns (Hudson and Lumb, 1996) with an important dlPAG connection involved in the modulation of endocannabinoid stress analgesia (Suplita et al., 2005). Early (1 and 3 days) and transient activation of microglia and prolonged reaction of astrocytes (14 days) were found in RVM in relation to long-lasting mechanical hyperalgesia after CCI of the rat infraorbital nerve (Wei et al., 2008). In contrast, a bilateral increase of astrocyte activation was demonstrated after 3 days with a decrease by day 10 following spinal nerve ligation (SNL). Conversely, although a weak immunofluorescence was observed at day 3, markedly stronger OX42 immunostaining was found at day 10 in rats operated on SNL (Leong et al., 2011). In our experiments, both types of sciatic nerve injury induced a strong activation of astrocytes and microglial cells in RVM, but no differences were found between the astrocyte- and the microglial activation induced by sCCI and CSNT. The results indicated that the intensity of glial activation in RVM after long–lasting nerve injury was not related to the extent of axotomy; this is consistent with the status of the RVM as a common output of descending modulatory system.

Retrograde neuronal death can be assumed as the initial signal for activation of glial cells after PNI (Flügel et al., 2001), but we did not investigate neuronal loss of RVM neurons in our experimental animals in relation to glial activation. However, there is some controversy about neuronal loss in RVM in different NPP models. While neuronal loss was found in RVM following spinal nerve ligature (Leong et al., 2011), it was absent after CCI or spared nerve injury (SNI) of the sciatic nerve (Leong et al., 2016). Therefore, other mechanisms of glial activation should be considered. Since CCL2 has a pivotal role in microglial cell activation in the dorsal horn after PNI (Zhang and De Koninck, 2006; Thacker et al., 2009; Van Steenwinckel et al., 2011; Clark et al., 2013), it is worth exploring the critical role of this chemokine also on supraspinal glial cross-talk which may exert NPP facilitation after sciatic nerve injury.

### Expression of CCL2 and CCR2 in Activated Glial Cells of PAG and RVM

Activated glial cells upregulate synthesis and secretion of numerous cytokines and chemokines that are involved in the exchange of signals between neurons and the glial cells of CNS structures. It was also demonstrated that these inflammatory mediators modulate nociceptive transmission and may influence NPP induction (DeLeo et al., 1996; Coyle, 1998; Colburn et al., 1999; DeLeo and Yezierski, 2001; Watkins et al., 2003).

Several lines of evidence suggest that CCL2 and its receptor CCR2 are expressed in both neurons and glial cells in the dorsal horn of the spinal cord in animal NPP models (White et al., 2005; Dansereau et al., 2008; Abbadie et al., 2009; Jung et al., 2009). CCL2 was detected in primary sensory neurons and their afferents in the dorsal horn of the spinal cord (Tanaka et al., 2004; Dansereau et al., 2008; Jeon et al., 2009). Besides primary afferents, CCL2 protein was also found in GFAP+ astrocytes activated by SNL (Gao et al., 2009). In contrast, immunohistochemical staining localized the CCR2 protein in spinal microglia after partial sciatic nerve injury (Abbadie et al., 2003), whereas the CCR2 mRNA signal was also present in deep dorsal horn neurons 3 days after SNL (Gao et al., 2009). Upregulation of CCL2/CCR2 in the spinal dorsal horn of NPP models suggests an important role for signaling through this chemokine in the modulation of chronic pain induction (reviewed in White et al., 2007; Gosselin et al., 2008; White and Miller, 2010). This was also confirmed by attenuation of behavioral signs of NPP in CCR2 knock-out mice (Abbadie et al., 2003).

With respect to surpraspinal structures, neuronal CCL2 and its receptor CCR2 associated with microglia were selectively upregulated in the RVM following SNL. Furthermore, injection of CCL2 into the RVM induced a dose-dependent hyperalgesia that was prevented by pretreatment with the CCL2 inhibitor RS-102895 (Guo et al., 2012). This showed that increased levels of CCL2 in RVM are related with descending facilitation of NPP. However, the precise cellular distribution of CCL2/CCR2 in PAG following PNI still remains unclear.

Our results revealed that neurons and activated astrocytes in both PAG and RVM displayed immunostaining for CCL2 protein, while activated microglial cells expressed CCR2 after chronic sciatic nerve injury. The results presented here and previously published studies of the cellular distribution of CCL2 and CCR2 suggest that CCL2/CCR2 signaling is involved in the neuron-glia and glia-glia interactions in both PAG and RVM after long-lasting nerve injury related to persistent pain. Based on the observed earlier activation of microglia, i.e., preceding astrocyte activation (Zhang and De Koninck, 2006; Hu et al., 2007), we can assume that the injury-associated nociceptive inputs trigger neuronal responses in PAG and RVM. Among these responses is the release of neuronal CCL2 that binds to CCR2 of microglia and promotes their activation (Zhang and De Koninck, 2006). Activated microglia may further stimulate astroglial activation through IL-18 and increase neuronal activity via CXCL1 (Abbadie et al., 2009; Gao and Ji, 2010). Furthermore, activated astrocytes may contribute to increased levels of CCL2 resulting in the augmentation of descending pain facilitation (Guo et al., 2012).

## Conclusion

Partial sciatic nerve injury by sCCI results in early and more distinct mechanical and thermal hypersensitivity up to 3 weeks. While CSNT induced a robust activation of astrocytes in both PAG and RVM, the activation of microglial cells was similar in these supraspinal structures after both types of sciatic nerve injury. This suggests that activated astrocytes in PAG and RVM may sustain the facilitation of the descending system to maintain NPP states, while activation of microglial cells may be associated with the reaction to long-lasting PNI. Furthermore, CCL2/CCR2 signaling may be involved in the neuron-glia and glia-glia interactions in both PAG and RVM after either sCCI or CSNT, triggering persistent NPP after PNI.

## Author Contributions

PD conceived, designed and coordinated the experiments, ensured quantitative immunohistochemical and western blot analyses and wrote the manuscript. PB-V conceived, designed and coordinated the study and wrote the manuscript. IK, IH-S and MJ designed and performed the experiments and also participated in acquiring and analyzing the presented data. All authors have approved the final version for publication.

## Conflict of Interest Statement

The authors declare that the research was conducted in the absence of any commercial or financial relationships that could be construed as a potential conflict of interest.
